# Normal fetal cardiac deformation values in pregnancy; a prospective cohort study protocol

**DOI:** 10.1186/s12884-019-2662-3

**Published:** 2019-12-27

**Authors:** Noortje H. M. van Oostrum, S. Guid Oei, Judith O. E. H. van Laar

**Affiliations:** Máxima Medical Centre, De Run, 4600 5504 Veldhoven, DB The Netherlands

**Keywords:** Speckle tracking, Fetus, Cardiac deformation values, Strain, Strain rate, Ultrasound

## Abstract

**Background:**

Myocardial deformation imaging offers the potential to measure myocardial function. Remodelling, the change in size, shape and function, appears as a result of pressure or volume changes and is thought to be the first sign of fetal adaptation to placental dysfunction. Deformation can be measured using speckle tracking echocardiography (STE). STE in the fetus might be useful for detection and follow up of the fetus endangered by placental dysfunction. Reference values for fetal myocardial deformation during gestation have not been comprehensively described and need further investigation before STE can be introduced in daily clinical practice. The aim of this study is to determine reference values for fetal myocardial deformation throughout gestation in uncomplicated pregnancies.

**Methods:**

A longitudinal cohort will be performed. 150 Women, pregnant from a non-anomalous singleton, will be included from 19 to 21 + 6 weeks gestational age. Thereafter, fetal heart ultrasounds will be performed 4 weekly, until 41 weeks gestational age or delivery. Ultrasound data will be analysed using STE software to determine reference values for fetal cardiac deformation during gestation.

**Discussion:**

Measuring cardiac deformation changes in pregnancy can be a promising tool to detect preclinical cardiac adaptation to placental dysfunction. However, previous studies used different ultrasound scans and STE software resulting in incomparable and contradictory results on deformation values. In this prospective study reference values during pregnancy, cardiac deformation values will be assessed with the same ultrasound and software package in 150 uncomplicated pregnancies.

**Trial registration:**

National Trial Register number: NTR7132. Date of inclusion: 2018/04/06.

## Background

Ultrasound has emerged as one of the mainstay examinations for fetal well-being. An important ultrasound examination to assess fetal well-being is fetal cardiac assessment. Pregnancies complicated by fetal growth restriction (FGR), maternal hypertensive disease and gestational diabetes (GDM) are often associated with placental dysfunction. Placental dysfunction endangers the normal growth and development of the fetus. The heart is a key organ in the process of fetal adaptation to hypoxia and under nutrition due to placental insufficiency. As a reaction to the chronic hypoxic environment and elevated placental resistance, resulting in pressure changes and volume overload of the fetal heart, the heart will start to remodel [1, 2]. The ventricle shape changes from an ellipse to a more globular shape and myocardial hypertrophy appears [2]. Both responses are an attempt to maintain stroke volume when hypoxia is present. The assessment of the cardiac function is therefore essential to the evaluation and/or treatment of complicated pregnancies. Currently, the assessment of the fetal and placental condition is performed by Doppler ultrasound examinations of the heart, and indirectly measured by the umbilical and cerebri media artery Dopplers. However, those traditional ultrasound measurement tools are often imprecise, angle dependent, subjective and become aberrant relatively late in the process of placental dysfunction. Speckle tracking echocardiography (STE) offers the advantage of non-Doppler measurement of myocardial deformation values as strain and strain rate. Speckle tracking has the advantage of being angle-independent and it does not require additional imaging as it uses the routinely acquired 4 chamber views of the fetal heart. Cardiac remodelling is thought to be detectable by ultrasound earlier in the process of fetal adaptation to placental dysfunction compared to the traditional heart function measurement [3, 4]. Therefore, early detection of fetal heart remodelling might be useful for early detection of placental dysfunction. Cruz-Lemini et al. [3] showed that fetal cardiovascular changes in FGR fetuses were still detectable at the age of 6 months, even without Doppler abnormalities. This suggests that prenatal cardiovascular remodelling remains after birth and might thus increases the risk of cardiovascular disease later in life. In addition, after fetal cardiac remodelling is shown, follow up measurements might be useful to monitor the placental function during pregnancy. This might improve the timing of delivery, before fetal deterioration appears, which probably results in improved perinatal and long term outcome.

STE offers the potential to measure early cardiac deformation, a direct measurement of remodelling and cardiac function. It is performed by an offline examination of the function of the myocardium using a 4 chamber view heart cycle clip. The STE software permits quantification of myocardial deformation using frame-by frame tracking of bright myocardial areas, speckles. Speckles are created by interference of ultrasound beams in the myocardium. The constructive and destructive interfaces is caused by back-scattering from structures smaller than the wavelength of ultrasound [5]. This results in small segments of the myocardium with stable and unique speckle patterns. Those patterns remain temporarily stable and can be traced from frame to frame. By tracking several points on the heart wall and calculating the shortening/lengthening between those points, deformation values can be calculated. In adults, several studies have investigated the relationship between deformation values and clinical outcomes. An association between deformation values, myocardial dysfunction and mortality in various pathological cardiac conditions was demonstrated [6–8].

Until today, the normal development of cardiac function throughout pregnancy in the fetus is not comprehensively described. Previous studies used different parameters for cardiac deformation [9–17], suboptimal study designs [11–17] and the reported results were contradictory.

STE is a promising tool to diagnose early cardiac deformation due to placental dysfunction in the fetus. However, before STE can be used in clinical practice, normal cardiac development throughout gestation has to be evaluated. Therefore, it is necessary to perform a prospective cohort study on normal strain and strain rate values throughout gestation.

## Methods/design

### Aim

The aim of this study is to determine reference values for the fetal myocardial deformation parameters strain and strain rate in healthy fetuses during gestation.

### Study design

A prospective cohort study will be performed. This study is approved by the medical ethical committee of the Máxima Medical Centre, Veldhoven, The Netherlands (NL64999.015.18).

### Setting

Fetal heart ultrasounds are performed at the Máxima Medical Centre, Veldhoven, The Netherlands. The Máxima Medical Centre is a tertiary care teaching hospital for obstetrics. The fetal heart ultrasounds are performed by certified and experienced sonographers.

### Participants

Women with an uneventful singleton pregnancy will be included in the study after written consent. The second trimester anomaly scan should be without any congenital anomalies that could possibly interfere with cardiac function. Patients will be included from 19 to 21 + 6 weeks gestational age. Women must be at least 18 years old.

Exclusion criteria are multiple pregnancies, insufficient understanding of the Dutch language, fetal arrhythmia, any suspicion of fetal congenital anomalies that might influence fetal cardiac function, pre-existing maternal hypertensive disease and auto-immune disease including SLE or Diabetes Mellitus.

Women who develop fetal growth restriction, hypertensive disease or gestational diabetes after inclusion will be excluded from the cohort.

10 Weeks postpartum information about the delivery and neonatal parameters as birth weight and Apgar score will be collected from the primary caregiver. If the neonate turns out to have any unexpected congenital anomalies that might influence fetal cardiac function, the patient will be excluded from the cohort.

### Sample size

To obtain normal values and 95% confidence intervals of healthy fetuses a study population of 100 uneventful pregnancies is needed [18]. 25 Participants are expected to be excluded due to pregnancy complications that appear after inclusion. Namely, 7–10% of uncomplicated pregnancies become complicated by FGR, 7–10% by maternal hypertensive disease and in 3–5% gestational diabetes will be diagnosed. This implicates that 10 participants are expected to be excluded due to fetal growth restriction, 10 due to hypertensive disease and 5 due to GDM. Anticipating on 25% insufficient data [19–22], another 25 participants will be excluded from the cohort. Therefore, to obtain 100 healthy participants in the cohort, 150 participants will be included initially.

### Procedures

Conduction gel will be applied to the women’s abdomen to perform the fetal heart ultrasound scan. The ultrasound is performed with a Philips EPIQ 7 W ultrasound system (Philips, Eindhoven, The Netherlands) by an experienced sonographer. The ultrasound setting used is standardised for fetal heart echocardiography. The pregnant woman will be lying down in a semi-recumbent position to prevent aortocaval compression. The fetal heart ultrasounds will be performed every 4 weeks. The ultrasound examinations will start from the moment of inclusion. Women will be included from 19 to 21 + 6 weeks gestational age. The ultrasound follow up measurements will be performed until 41 weeks gestational age or delivery.

After the four chamber view is visualized, 3 to 6 DICOM clips of at least 3 heart cycles will be performed and stored offline. The DICOMs are acquired during fetal rest, with the pregnant woman holding her breath for a few seconds, to avoid as much artefacts as possible. The stored DICOM data will be examined offline using a fetal cardiac speckle tracking software program (2D Cardiac Performance 1.2) developed by TomTec Imaging Systems GmbH (Munich, Germany). To identify the beginning and end of a cardiac cycle, the anatomic M-Mode feature in the software will be used. A line will be drawn across the left ventricular wall through the ventricle and the septum. A corresponding M-Mode appears. The R waves in the M-Mode correspond to the opening and closure of the mitral valves and will be used to identify one heart cycle. After, the endocardial border for each ventricle will be traced from the junction of the lateral wall annulus to the apex and from the apex to the base of the junction of the septal wall annulus. After the tracing, an automated analysis detects the endocardial border during 1 heart cycle providing fetal cardiac deformation values. The same heart cycle in the same DICOM clip will be examined twice, preventing issues with the repeatability within consecutive cardiac cycles.

The obtained fetal cardiac deformation values will be used to determine reference values.

### Study parameters

The fetal myocardial deformation parameters that will be obtained are:

Strain (%): s*train represents myocardial deformation in response to an applied force.*

Strain rate (1/strain): *Strain rate describes the rate of deformation as a percentage of the original dimension.*

### Statistical analysis

The collected data will be analysed using SPSS_22. Several analysis will be performed.

Analysis of the images will be performed offline by 2 different researchers who are blinded for each other’s results. The images will be examined twice by the same examiner, leaving 4 weeks in between, so the examiner will be blinded for the first results. Inter-and intra-observer variability will be obtained by calculating intraclass correlation coefficients (ICC). Limits of agreement will be assessed using Bland-Altman analysis.

A linear mixed model analysis will be used to determine reference values of fetal cardiac deformation during gestation.

## Discussion

Previous studies have been published regarding normal values for strain and strain rate in the fetus during gestation using speckle tracking [9–17]. Cardiac deformation imaging in the fetus is still experimental. Di Salvo et al. [21] and Krause et al. [19] showed that STE is a feasible and reproducible approach to assess fetal cardiac function. However, contradictory findings of cardiac function values are shown. This can be explained by differences in STE software that was used, study limitations as cross sectional study design, low number of included fetuses and low frame rates in the heart cycle clips [23]. Even more, in the fetus, some technical challenges while using speckle tracking remain to be solved. An ECG cannot be performed synchronised with the ultrasound of the fetal heart as it is performed in adults. A simultaneously performed fetal ECG and ultrasound could possibly improve the reliability and reproducibility of the measurements. Next to technical challenges, functional cardiac evaluation in the fetus using speckle tracking remains difficult, as the quality of the imaging is dependent on fetal movements, fetal position, fetal heart rate, maternal movements and maternal habitus. Suboptimal imaging might cause suboptimal contrast between the ventricular cavity and the myocardial wall resulting in poor tracking of the endocardial border.

STE in the fetus can be easily performed from early gestation, is non-invasive, non-expensive and easy to apply in already existing daily practice. Fetal heart deformation is thought to be the first sign of fetal adaptation to impaired placental function. Therefore, the measurement of fetal heart deformation using STE could be a promising clinical tool in the daily follow up of pregnancies complicated by placental dysfunction. Preclinical deformation of the fetal heart could help to distinguish the endangered fetus from a constitutional small fetus.

However, before STE can be used as a clinical tool in complicated pregnancies, the establishment of normal strain and strain rate reference values in a healthy cohort during gestation is mandatory, prerequisite for its use in evaluating (pathologic) changes of function of both ventricles during pregnancy. There is a need for age specific reference values, assessed using the same ultrasound and software package, in a longitudinal study design, for the adequate interpretation of measurements of STE.

To obtain reliable reference values during pregnancy, this study will assess cardiac deformation values with the same ultrasound and software package in 100 uncomplicated pregnancies. These reference values are mandatory before cardiac deformation values in complicated pregnancies can be evaluated for clinical use.

Increased risk in cardiovascular disease during lifetime is expected in children born from a pregnancy complicated by placental dysfunction. In the future, for follow up, neonatal and infant growth and development might be examined to assess the long-term consequences of fetal cardiac remodelling.

## Data Availability

The raw datasets used and/or analysed during the current study are freely available from the corresponding author on reasonable request to any scientist wishing to use them for non-commercial purposes, without breaching participants’ confidentiality.

## Abbreviations

FGR: Fetal growth restriction
GDM: Gestational diabetes
SLE: Systemic lupus erythematosus
STE: Speckle tracking echocardiography
